# A Simplified Supine Technique Expedites the Delivery of Effective Craniospinal Radiation to Medulloblastoma – Comparison with Other Techniques in the Literature

**DOI:** 10.7759/cureus.404

**Published:** 2015-12-15

**Authors:** Patricia Tai, Rashmi Koul, Khanh Vu, Trent Edwards, Joseph Buwembo, Alisson R Teles, Muhammad Salim

**Affiliations:** 1 Department of Radiation Oncology, Allan Blair Cancer Center, University of Saskatchewan; 2 Radiation Oncology, CancerCare Manitoba, University of Manitoba; 3 Department of Radiation Oncology, Maisonneuve-Rosemont Hospital; 4 Department of Neurosurgery, Regina General Hospital; 5 Department of Neurosurgery / Laboratory of Clinical Studies and Basic Models of Spinal Disorders, Universidade de Caxias do Sul; 6 Department of Medical Oncology, Allan Blair Cancer Center, University of Saskatchewan

**Keywords:** medulloblastoma, radiotherapy, therapy, posterior fossa syndrome

## Abstract

A 28-year-old man presented to the emergency room with a severe headache of one day's duration. A computerized tomography scan showed a hemorrhagic tumor measuring 3.9 x 4.4 cm in the left cerebellar hemisphere. The resection specimen revealed medulloblastoma. He had two episodes of rebleeding and multiple postoperative issues preventing the use of prone craniospinal radiotherapy. We designed a supine technique for this tall man, which was not complicated to set up. The rapid safe implementation of this technique allowed us to avoid further rebleeding and successfully treat the residual tumor. This technique is the described technique in this case report and is compared to other techniques. At 7.5 years after surgery, he is alive without cancer and with only a mild residual deficit. This case is unusual since the majority of patients with the diagnosis of hemorrhagic medulloblastoma died.

## Introduction

Craniospinal irradiation (CSI) is used in neoplasms with the risk of spread to the craniospinal space, such as medulloblastoma, central nervous system (CNS) leukemia, germ cell tumors, high-grade ependymomas, and multicentric CNS lymphomas. For many decades until around 2005, the CSI technique used a prone position throughout the simulation and treatment. Children or non-compliant adults often find it difficult to stay in the treatment position for the required time. The case below illustrates the successful management of a hemorrhagic medulloblastoma using an alternative simplified set-up method. Informed patient consent was obtained at the time of initial surgical and radiotherapy treatment. The patient kindly consented to allow us to write up his interesting journey of nearly full recovery per policy of our University.

## Case presentation

The patient was a previously healthy 28-year-old man who presented to the emergency room in the spring of 2008 with a one-day history of a headache after shoveling snow. It resolved but recurred the next day. His headache became very severe with subsequent nausea and vomiting. Computerized tomography (CT) scan at 15:10 hours showed a hemorrhagic tumor measuring 3.9 x 4.4 cm in the left cerebellar hemisphere, with cerebellar tonsillar herniation and midline shift. While in the emergency room, he developed a decreasing level of consciousness and apnea, requiring intubation and ventilation. The repeat CT at 23:20 hours demonstrated more bleeding and the tumor measured 6.3 cm. Therefore, at midnight he underwent an emergency frontal ventriculostomy, left suboccipital craniectomy, evacuation of hematoma, and tumor debulking. The next day, a repeat CT showed a residual tumor mass measuring 3.5 x 3.1 cm. Since the residual was more than 1.5 cc, the stage was considered to be a high-risk disease. Edema was present in the deep left cerebellar hemisphere, and there was slight compression and shift of the midline, including some compromise of the fourth ventricle. There was some persistent mass effect upon the left posterior brainstem and quadrigeminal plate cisterns were effaced.           

He suffered a third episode of hemorrhage into the tumor bed with increased intracranial pressure on postoperative day (D) 4. He was re-operated to evacuate the hematoma. At that time, a portion of the cerebellum had become necrotic and swollen and was therefore evacuated. He underwent a third craniotomy on D12 to evacuate a new hemorrhage in the tumor bed, i.e. his fourth episode of bleeding. Pathology confirmed a Grade IV medulloblastoma.

His clinical course continued to be very unstable, necessitating that he remained in the intensive care unit with sepsis and fever, although cultures were negative. He had respiratory failure and was ventilated for 21 days. He required a tracheostomy. He was suffering from a persisting severe productive cough with excessive secretions, requiring frequent suctioning. His blood pressure was labile, with systolic varying between 100 to 180 mm Hg. He had a decreased level of consciousness, with fluctuating Glasgow coma scale of 8-10. Some days he did not respond to commands. He also developed agitation, seizures, and intolerance of tube feeding with continual vomiting. A repeat CT scan following surgery still showed persistent blood and edema in the cerebellar area. His severe weakness, the decreased level of consciousness, and poor general condition were most likely due to posterior fossa syndrome.

On D31 postoperatively, his level of consciousness improved after dexamethasone was increased in frequency from 4 mg thrice to four times a day. He could obey commands to open his eyes and move the toes. He had limited facial expression. He was on enoxaparin, 40 mg subcutaneously, to prevent deep vein thrombosis that, fortunately, did not provoke cranial rebleeding. The cerebellar hematoma was smaller on the MRI scan on D43 compared to that on D32.

**Figure 1:**
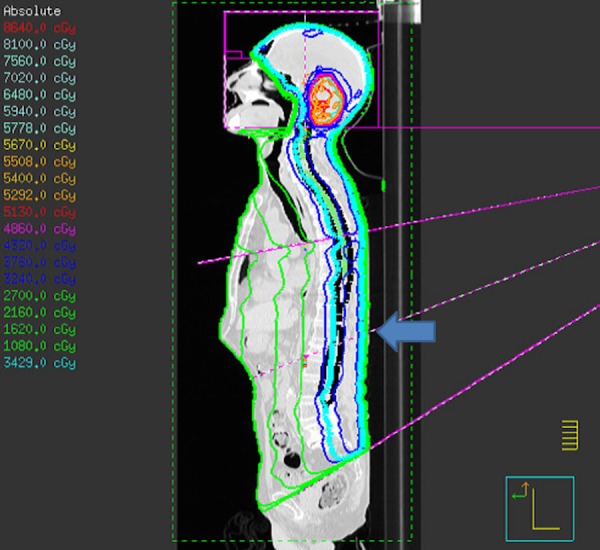
Our supine craniospinal irradiation (CSI) technique Cranial and superior spine fields use a half-beam block without skin gap. The isocenters of both fields were at mid-cervical level. The inferior spine field was centered on the first lumbar vertebra, marked by a blue arrow. Its upper border matched the diverging lower border of the superior spine field at the sixth thoracic vertebra. The dose was homogeneous within the target without any hot spots. After 36 Gy/20 fractions of CSI, a boost 18 Gy/9 fractions was delivered to the posterior fossa.

A prone CSI technique would have been impossible because of his moderate paroxysmal coughing spells, secretion from the tracheostomy requiring frequent suction, and oxygen. Our department designed a relatively simple supine CSI technique without skin gaps to facilitate treatment (Figure 1).

There was no skin gap between the cranial and upper spinal fields since both had half-beam blocks. The half-beam block isocenter was just below the cranial planning target volume (PTV). It would have been more ideal to position it further inferiorly if the field size limitation (20 cm) allowed the entire head to be covered with margin. Therefore, the position of the neck junction depends on the size of patient’s head. For a smaller head, the junction can be in the lower neck. Imaging is done for brain/superior spine fields (which are mono-isocentric) and to ensure the patient is straight per length of the treatment area.    

Both spinal fields have couch rotation to 270 degrees. The patient was shifted superior-inferiorly. The couch was “kicked” through to 270 degrees and the gantry was rotated to match the divergence of the superior border of the inferior spine field to the inferior border of the superior spine field (imaging could not be done once the couch was “kicked”). The inferior border of the superior spine field was marked on the underside of the couch (paper and marker) for checking. The inferior spine field can be treated with extended skin-source distance (SSD) if required to treat the remainder of the spine. Stepped control points were used to smooth the dose over the length of the spine. The plan contains four junction shifts at 0.5 cm to smooth out any chance of over/underlap where fields match. All junctions were shifted weekly, i.e. every 9 Gy. Compensators were used. A cranial aquaplast shell and body vac lok were used for immobilization.

This technique did not require intensity modulation radiotherapy, which simplified and expedited his treatment planning, and quality assurance was less complicated. Planning CT was done on D36 and treatment started on D38 postoperatively. With this technique, the amount of time required at the time of CT simulation was not prolonged and the patient could remain still for the duration of each fractional treatment. The total set-up and treatment time was 40-50 minutes because he required suctioning of sputum. The supine technique was well tolerated and more stable than the prone technique and, as a consequence, this results in a reduced daily treatment time.

At the conclusion of chemotherapy, magnetic resonance imaging (MRI) head showed complete remission of the residual tumor. At last follow-up in the fall of 2015, he finished all physical rehabilitation and could now walk with minimal support. His speech was initially muffled and now he is comprehensible on the phone. He has diplopia and limited facial expression. Due to mild ataxia on examination, past-pointing is worse with the left hand than the right hand. He frequently enjoys watching his favorite football, sitting in the stands at the stadium. MRI scan of the head has not shown any recurrence in the posterior fossa. Our department has stopped the prone technique since this case and adopted this technique as a standard set-up.

## Discussion

The current standard treatment of medulloblastoma consists of maximal tumor resection followed by craniospinal radiotherapy and chemotherapy [1-2]. Over the years, multimodal treatment evolved and has had improved outcomes with around two-thirds of these patients being long-term survivors [3].

In general, the clinical presentation of medulloblastoma is truncal ataxia and progressive symptoms of intracranial hypertension due to obstructive hydrocephalus. Spontaneous hemorrhage is rarely observed in medulloblastoma, and it has been thought to occur as a result of upward herniation or vascular invasion by tumor cells [4]. In 2002, Fukai, et al. reviewed the literature and found 30 cases of hemorrhage associated with medulloblastoma [5]. The majority of reported cases were associated with external ventricular drainage, subtotal removal, or radiation [5-8]. Although uncommon, bleeding is often fatal. In a large study on intracranial hemorrhage of children with cancer, it was observed that only 3% of children with brain tumors experienced tumor bleeding, which was associated with a 50% mortality [9].  

This patient had sudden bleeding within the posterior fossa and underwent urgent ventriculostomy, tumoral debulking, and hematoma evacuation. The aims of surgery in medulloblastoma are maximum safe resection without compromising neurological function to reduce the mass effect, establish a diagnosis, and if possible, to restore cerebrospinal fluid flow. Unfortunately, the patient had more episodes of rebleeding in the tumor bed during the early postoperative period requiring surgical evacuations. Repetitive intratumoral hemorrhage is extremely rare [10].

After resection and stabilization, radiotherapy was started locally with the intent to reduce the risk of re-bleeding and future recurrence of the tumor. His clinical condition prevented a prone position for CSI, resulting in the development of this alternative CSI technique. Advantages of supine over prone CSI techniques are: (A) It is much better tolerated. The patient is more comfortable. The position is more stable and reproducible. Shielding of the cribriform plate and inferior temporal lobes is more accurate [11]. (B) It allows intubation for anesthesia, which may be required in young children (< 6 years of age), and reduces anesthetic complications due to alteration of cardiac function, including pulmonary blood flow [11]. (C) It is easier for patients with severe weakness and in poor general condition. Transfer to the treatment couch and management of the airway are both facilitated compared to prone CSI techniques [12]. (D) This technique requires only longitudinal couch motions and is simple to plan, is easy to incorporate into the workload of a busy radiotherapy department, and may be adapted to newer delivery techniques (arc-based therapy or other IMRT). (E) It significantly reduces the amount of time on the CT simulator table because the planning itself is performed after the patient has left the department. This is a major advantage for young patients who have recently undergone major surgery to promote patient comfort.

Our technique, using half-beam blocked fields on both cranial and superior spine fields, is like the single isocentric head and neck technique, which therapists are comfortable to perform. The disadvantages of this technique are (A) divergence into the pelvis resulting in more direct or scattered doses to the gonads. Although ovaries can be pinned laparoscopically to avoid the radiation beam by pediatric surgeons, these transplanted ovaries may not function normally in future. Other techniques use an asymmetric jaw to sharply cut off the inferior field border. (B) It also requires the use of anterior reference points and meticulous documentation of shifts to ensure the match is occurring properly as visualization of skin gaps for matching is not possible, unlike prone techniques. We used portal imaging and marks on the bottom of treatment couch to check matching. (C) Cranial field size is limited due to the maximum dimension of 20 cm with a half-beam block unless extended skin-to-axis distance (SAD) is used. Therefore, the planning target volume is close to the inferior border of the cranial field (Table 1).    

**Table 1:** Comparison of different craniospinal techniques D: dimensional IMRT: intensity-modulated radiation therapy Inf: inferior Sup: superior

	Alternatives	Pros vs. Cons
Machine	(1) photon, (2) electron, (3) proton	(1) Simple. (2) Less dose at depth, more complicated matching and sparing may be less for high energy electron. (3) Less dose at depth and risk of a second malignancy, but limited locations of proton facilities.
Position	(1) prone, (2) supine	See Discussion section. Supine technique has many advantages.
Planning & junctions	Planning: (1) 2D, (2) 3D, (3) IMRT, helical tomotherapy Junctions: Shifting junctions, half-beam block, skin gap and matching 50% isodose at spine, feather the match, overlap fields which have a gradient at match	(1) Simple but requires junction shifts. (2) Narrower spinal fields but more labor intense contouring and planning. (3) Less dose at depth, no need for junction shifts by intensity modulation at match lines but concerns of larger low-dose region areas with higher risk of second malignancy, more labor intense contouring planning and quality assurance.
Cranial field	(1) Isocentre fixed in: (1a) mid-brain, (1b) neck junction (half-beam block). (2) Collimator angle: (2a) matches superior spine field divergence. (2b) not required if neck junction is vertical using half-beam block in superior spine field.	(1a) Same shielding block in the cranial field but needs junction shift. (1b) No couch angle for superior spine field and no junction shift. (2b) Simpler to treat as in our case.
Superior spine field	(1) Neck junction: (1a) couch angle to match cranial field divergence. (1b) half beam block. (2) Isocentre at (2a) thoracic spine: superior border matches cranial field, (2b) neck junction: half-beam block. (3) Gap(s) with cranial and inferior spine field.	(1a) Junction shifts required. (1b) No couch angle/junction shifts, faster. (2a) Larger superior spine field possible and for some may be able to use a single field, but junction shifts required. (2b) Used in our case, simpler but limits the size of superior field, increased divergence at inferior edge. Hard to verify visually with supine positioning.
Inferior spine field	(1) Superior border match superior spine field divergence. (2) Spade expansion to cover nerve root disease, if suspected	(1) Used in our case, simpler but portal imaging required

The table summarizes and compares different published CSI techniques in the literature [11-24]. Of note, Parker, et al. found IMRT did not significantly increase the integral dose [26], despite common belief. Bauman, et al. described the use of a posterior spinal wedge pair to avoid direct irradiation of the unhealed wound [23]. While proton CSI has an ideal dosimetry, the limited number of proton facilities results in lost revenue and clinical trial recruitment for the referring center. The patient and caregiver are often required to travel long distances. Rapid referral is necessary for medulloblastoma to meet the standard of start date within 31 days postoperatively, which is impossible for patients suffering from postoperative sequelae. Our patient had posterior fossa syndrome [27] and was not accepted by other centers in 2008. Fortunately, he has recovered with minimal sequelae over a period of several years. This noteworthy and unusual case illustrates that a simplified supine CSI, which expedited the postoperative management of hemorrhagic medulloblastoma, can be employed in other similar circumstances.

## Conclusions

We have successfully treated a young adult with a medulloblastoma, which presented with sudden deterioration due to acute tumor bleeding. He had four episodes of bleeding. We designed a relatively simple supine CSI technique for this difficult case, and we also found that it reduces simulation and set-up times. It was successfully implemented to prevent further rebleeding. After 7.5 years’ follow-up, he remains free of disease with only a mild neurological deficit and enjoys an excellent quality of life.
